# Nuclear and Mitochondrial Genome Assemblies of the Beetle, *Zygogramma bicolorata*, a Globally Important Biocontrol Agent of Invasive Weed *Parthenium hysterophorus*

**DOI:** 10.1093/gbe/evad188

**Published:** 2023-10-13

**Authors:** Ranjit Kumar Sahoo, Shivakumara Manu, Naveen Kumar Chandrakumaran, Karthikeyan Vasudevan

**Affiliations:** Laboratory for the Conservation of Endangered Species (LaCONES), CSIR-Centre for Cellular and Molecular Biology (CCMB), Hyderabad, India; Laboratory for the Conservation of Endangered Species (LaCONES), CSIR-Centre for Cellular and Molecular Biology (CCMB), Hyderabad, India; Laboratory for the Conservation of Endangered Species (LaCONES), CSIR-Centre for Cellular and Molecular Biology (CCMB), Hyderabad, India; Laboratory for the Conservation of Endangered Species (LaCONES), CSIR-Centre for Cellular and Molecular Biology (CCMB), Hyderabad, India

**Keywords:** biological control, Chrysomelidae, leaf-feeding beetle, reference genome, NUMTs, phylogeny

## Abstract

Implementing a genetic-based approach to achieve the full potential of classical biocontrol programs has been advocated for decades. The availability of genome-level information brings the opportunity to scrutinize biocontrol traits for their efficacy and evolvability. However, implementation of this advocacy remains limited to few instances. Biocontrol of a globally noxious weed, *Parthenium hysterophorus*, by the leaf-feeding beetle, *Zygogramma bicolorata*, has been in place for more than four decades now, with varying levels of success. As the first step in providing genetic-based improvement to this biocontrol program, we describe the nuclear and mitochondrial assemblies of *Z. bicolorata*. We assembled the genome from the long-read sequence data, error corrected with high-throughput short reads and checked for contaminants and sequence duplication to produce a 936 Mb nuclear genome. With 96.5% Benchmarking Universal Single-Copy Orthologs completeness and the long terminal repeat assembly index 12.91, we present a reference-quality assembly that appeared to be repeat rich at 62.7% genome-wide and consists of 29,437 protein-coding regions. We detected signature of nuclear insertion of mitochondrial fragments in 80 nuclear positions comprising 13 kb out of 17.9 kb mitochondria genome sequence. This genome, along with its annotations, provides a valuable resource to gain further insights into the biocontrol traits of *Z. bicolorata* for improving the control of the invasive weed *P. hysterophorus*.

**Significance**

Leaf-feeding beetles not only comprise a species-rich group, but many of these species are also designated as pests or biocontrol agent. *Zygogramma bicolorata*, in particular, checks the spread of the obnoxious weed *Parthenium hysterophorus*. By describing the whole genome of *Z. bicolorata*, our current study paves the way for further investigations targeting biocontrol efficiency in the species; for instance, through breeding design or marker-assisted population monitoring. Overall, this genome will be a valuable resource for research in NUMTs, phylogenomics, and comparative genomics.

## Introduction

Classical biocontrol by introducing coevolved exotic natural enemies to manage invasive plant species is often advocated over conventional management strategies, primarily because of the cost-effectiveness and environmentally benign nature of the biocontrol programs (Thomas and Willis 1998; Messing and Wright 2006; Seastedt 2015). However, biocontrol attempts do not always provide adequate outcomes due to a variety of reasons (Stiling 1993; Myers 2000; Thomas and Reid 2007). For instance, over an ecological time scale, the introduced natural enemy may establish undesirable interactions with the nontarget species (Louda et al. 1997; Driesche and Hoddle 2016; He et al. 2021), including the disease-causing agents (Pearson and Callaway 2006). In another instance, the climatic variations may favor the invasive weed (Sun et al. 2022) and result in a mismatched distribution between the weed and its biocontrol agent (Harms et al. 2021) or break the synchronization in their life cycles as well (Stiling 1993). As a consequence, the biocontrol effectiveness remains below par. Although the prerelease quarantine protocol ascertains host specificity, damage potential, and population growth propensity of the biocontrol agent, the underlying genetic variations and their evolvability remain obscure, which otherwise would facilitate improving the efficacy of the biocontrol strategies (Leung et al. 2020).

In augmenting the biocontrol program with genetic-based solutions, we present the first genome assembly of the leaf-feeding beetle *Zygogramma bicolorata* Pallister, 1953 (Coleoptera: Chrysomelidae), a biocontrol agent of global importance. Native to tropical America, *Z. bicolorata* has been introduced in many countries across Australia, Asia, and Africa (Dhileepan and Strathie 2009; Dhileepan and Wilmot Senaratne 2009; CABI 2021) to control the invasive plant species *Parthenium hysterophorus*, a weed known to cause extensive ecological and economic damage in its invasive range (Sushilkumar and Varshney 2010; Adkins and Shabbir 2014) and imparts severe health hazards in human and livestock as well (reviewed in Sushilkumar 2005). The release of *Z. bicolorata* appears to check *Parthenium* load by affecting plant growth, flower production, and the soil seed bank negatively (Jayanth and Ganga-Visalakshy 1996; Dhileepan et al. 2000). However, concerns remain about many other aspects of the biocontrol agent, for instance, the seasonal effect of its biocontrol efficiency (Dhileepan 2003), its restricted distribution relative to that of *P. hysterophorus* (Dhileepan and Wilmot Senaratne 2009; Gharde et al. 2019), and possible host plant expansion to include *Xanthium strumarium* (Kumar 1992; Sushilkumar and Bhan 1996), where genomic data are likely to enhance and accelerate further investigation.

Using Nanopore long-read sequencing data, we assembled the genome of *Z. bicolorata* and polished the assembly with high-throughput Illumina short reads. The assembly was checked for microbial contaminants and purged for sequence duplicates. Assessment of the final assembly for protein-coding orthologs and intact long terminal repeats (LTRs) indicated a reference-quality genome assembly. The phylogenetic position of *Z. bicolorata* was evaluated through tree reconstruction from protein orthologs. We annotated the repeat and coding regions of the genome to provide an overview of the genome content. The mitochondrial genome was assembled separately, and we detected the signature of nuclear fragments being homologous to the mitochondrial genome (nuclear insertion of mitochondrial fragments [NUMTs]). Overall, the availability of this genome would prompt comparative genomics to gain adequate knowledge of the traits that are determinants of biocontrol success and expedite population genetics to monitor the ecological response of the biocontrol agent in the introduced habitats (Leung et al. 2020).

## Results and Discussion

### Nuclear Genome Assembly

**Fig. 1.**
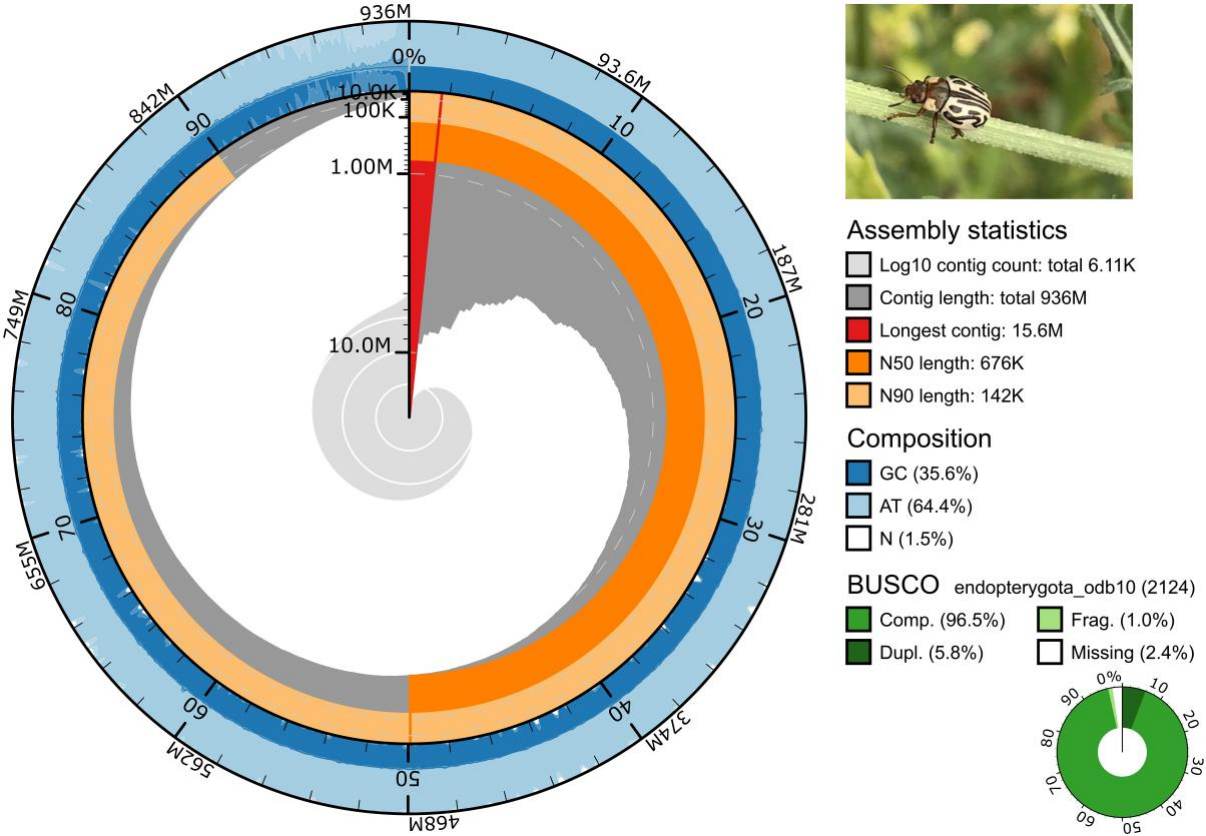
Nuclear genome assembly of *Zygogramma bicolorata*. The assembly snail plot along with the assembly statistics, sequence composition, and BUSCO comparisons.

**Fig. 2.**
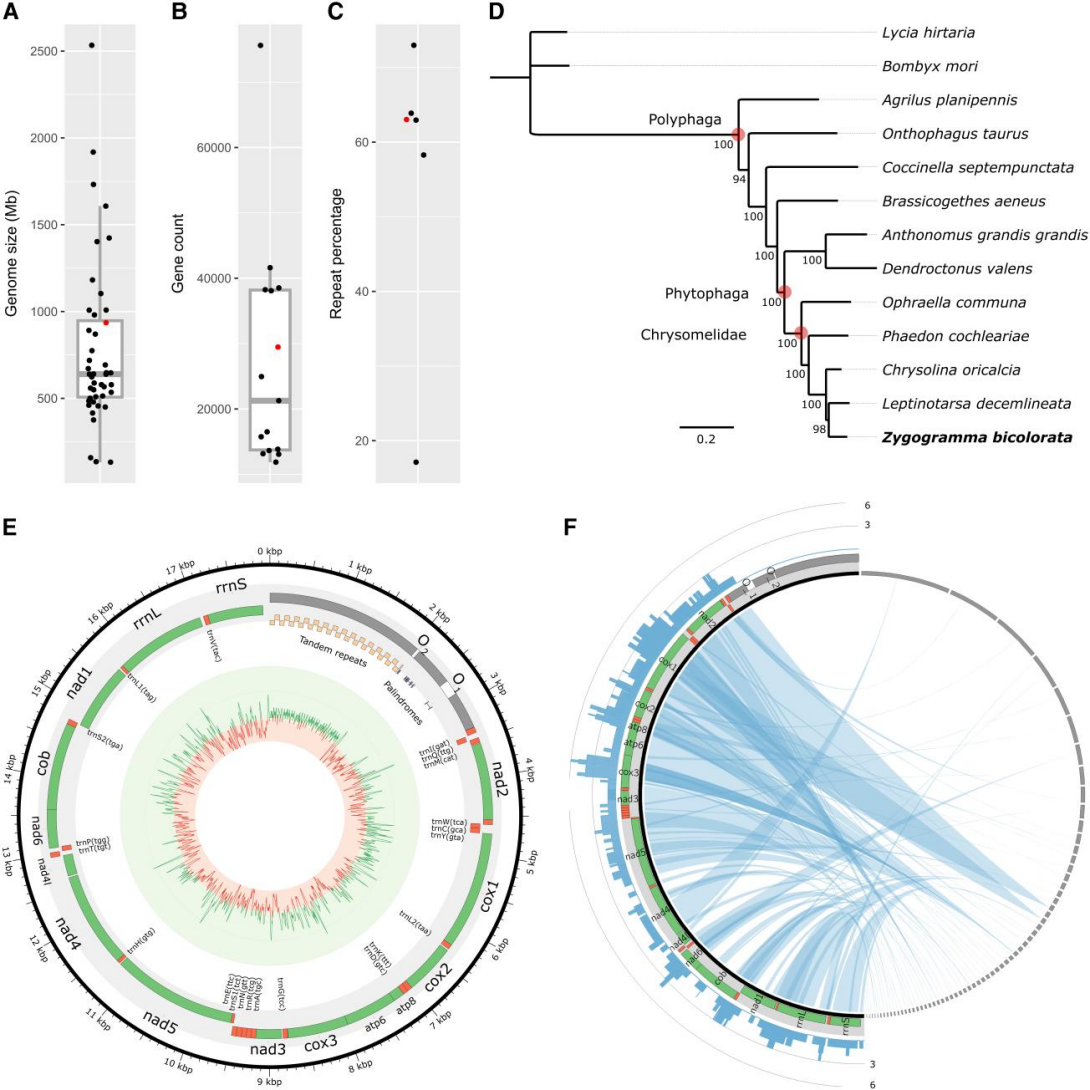
Distribution plots of Chrysomelidae genome statistics: (*A*) genome size, (*B*) gene count, and (*C*) percentage repeat elements. See Supplementary material for the underlying data. Data of *Zygogramma bicolorata*, from the current study, is colored red. (*D*) Consensus tree (log-likelihood −4,345,003.42) of selected beetle taxa, including *Z. bicolorata*, along with two moth species as outgroup. Node value indicates bootstrap support. Circled nodes correspond to the labeled clades. (*E*) Annotated mitochondrial genome plot. GC content graph is toward the center, where the outer boundary of the inner red circle represents the mitogenome-wide average. (*F*) NUMT distribution and frequency plot: the left-side continuous thick black line represents the mitochondrial genome and the right-side dashed gray lines represent nuclear contigs that harbor the NUMTs. Within the circle, the blue ribbon connects the NUMTs to the respective mitochondrial segment based on blast similarity (see Materials and Methods). The bar graph represents the base-level frequency (0–6 scale) of mitochondrial segments within the NUMT profile, indicating the number of times the mitochondrial fragments appear as NUMT.

Using error-corrected 121 Gb short-read and 31 Gb long-read data from a laboratory-inbred single female, we present the first assembled nuclear genome of *Z. bicolorata* of 936 Mb size contained within 6,106 contigs (fig. 1). The assembly size is within the expected value of 807–1,006 Mb that was estimated from the short-read-based *k*-mer analyses (supplementary fig. S1, Supplementary Material online) and falls toward the upper quartile of the assembled genome size distribution (132 Mb to 2.5 Gb) in the Chrysomelidae family (fig. 2*A*, supplementary table S5, Supplementary Material online). The assembled genome comprises 11 pairs of autosomes and 1 pair of sex chromosomes, as obtained from the species’ karyotype previously (Bhatti et al. 2017). Fifty percent of the genome is contained within 293 contigs in our assembly (L50), where the shortest contig is 676 kb (N50), and 163 contigs are longer than 1 Mb, with the longest being 16 Mb. The base-level consensus quality (QV), based on the *k*-mer survival rate, is 36.4 (error rate 0.00023) for the assembly, indicating a 99.9% accuracy in consensus calls. Gene space evaluation using Benchmarking Universal Single-Copy Orthologs (BUSCO) genes at the “Insecta” and “Endopterygota” levels predicts assembly completeness of 97.5% and 96.5%, respectively (fig. 1, supplementary table S1, Supplementary Material online). Furthermore, a comparison of intact and total LTRs in the assembly estimates the raw LTR assembly index (LAI; Ou et al. 2018) as 12.91, which falls within the LAI range of 10–20, indicating a reference-quality genome assembly.

Tree analysis using 669 BUSCO proteins common across 11 beetle species/genera places *Z. bicolorata* within the monophyletic clade Chrysomelidae (fig. 2*D*). We provide the first genome-wide support for the phylogenetic placement of *Z. bicolorata* in favor of the only investigation that had included *Z. bicolorata* along with two other chrysomelid genera to build a mitochondrial phylogeny using one locus (Yocum et al. 2011). Our phylogenetic reconstruction of Polyphaga is congruent with the taxon-rich analysis of 95 protein-coding genes (Zhang et al. 2018).

Our ab initio gene prediction results in 29,437 gene models and 32,265 transcripts, of which 19,585 transcripts (61%) were functionally annotated against the eggNOG database. The annotated gene regions show about 91% BUSCO completeness with 13% duplication against the “Insecta” orthologous database (supplementary table S1, Supplementary Material online). The number of gene models is comparable with the available annotations in other Chrysomelidae species, which range from 11,862 to 75,642 (fig. 2*B*, supplementary tables S3 and S4, Supplementary Material online). Yet, in *Z. bicolorata*, we observe a significant overlap between gene and repeat annotations: 8.4% of CDS and 75.2% of intron regions from the gene models overlap with the repeat elements (supplementary table S2, Supplementary Material online). Total annotated repeats in *Z. bicolorata* combined to 62.7% of the assembled genome, similar to other Chrysomelidae, except for the low repeat content in *Leptinotarsa decemlineata* (fig. 2*C*). DNA transposons are the major repeat group at 30.3% of the genome, and retroelements are 23.4%. Within retroelements, the LTR class has the highest abundance (20.4% of the genome), followed by LINEs (3.0%). SINEs are present in limited numbers by covering only 21,763 bp. Only 8.2% of the annotated repeats were not classified.

### Mitogenome Characterization and NUMTs

The mitochondrial genome assembly is 1 circular contig of 17.9 kb size, containing 13 protein-coding genes, 2 ribosomal RNAs, and 22 transfer RNAs (fig. 2*E*). Nine protein-coding genes and 14 tRNAs are present on the plus/major strand. This strand also harbors a 3.3 kb control region (CR) with 2 putative sites for the origin of replication/transcription. The origin sites are 42 and 198 bp long and separated by a 519 bp sequence. Seven palindromes or inverted repeats (IRs) are found dispersed across the origin sites. The arms of the IRs are 10–16 bp with the spacer region of 3–85 bp. Notably, the origin sites are A + T rich at an estimated 88% average. Contrastingly, the part of the CR that flanks upstream to the origin site is A + T poor, at 72.5% average, in comparison with the background content of 78.6% (fig. 2*E*). Interestingly, this A + T poor region harbors tandem repeats, where a 71 bp unit is repeated 29 times and a partial copy once (0.01% mismatch throughout). The occurrence of tandem repeats of different unit lengths has been characterized recently in other beetle species, for instance, a 250 bp repeat unit in *Meiligma* sp. and a 36 bp unit in *Euwallacea interjectus* (Guo et al. 2023; Huang et al. 2023).

We identified the signature of about 13 kb of the mitochondrial genome, excluding the low complex CR, being inserted into the nuclear genome at least once (fig. 2*F*). Nevertheless, the homologous mitochondrial segment of the longest NUMT, of 3,197 bp, extends to the low complex CR at 331 bp. The NUMTs are validated with evidences from long-read sequencing data, showing the read depth between 4X and 58X (supplementary fig. S4*A*, Supplementary Material online). Most NUMTs are of small size: 76 out of 80 total NUMTs were below 1 kb in length, 51 of which were <200 bp. Notably, there was no clear relationship between the NUMTs size (ranging 33–3197 bp long) and the sequence divergence in NUMTs versus mitochondrial homologs (ranging 71–100% identity; supplementary fig. S4*B*, Supplementary Material online).

## Conclusions

With high-throughput sequence data and up-to-date analytical approaches, we describe the first genome assembly of the biocontrol agent *Z. bicolorata* and confirm its phylogenetic position within the Chrysomelidae family. This assembly would serve as a primary resource to provide genetic-based solutions to the biocontrol problems in managing the spread of the noxious weed *P. hysterophorus* globally. Additionally, with the identification of NUMTs in *Z. bicolorata*, we advocate caution in the design of mitochondrial markers and their application in selective breeding or population monitoring.

## Materials and Methods

### Beetle Sample

Fifteen adult *Z. bicolorata* consisting of seven males and eight females sourced from Berhampur, India were laboratory reared on the fresh leaves of *P. hysterophorus* at ambient conditions. Freshly eclosed adult females from the third laboratory generation were food deprived for 2 days and subsequently preserved at −80 °C. Prior to DNA extraction, the preserved sample was surface sterilized with 95% ethanol followed by distilled water twice. The whole body of one adult female was used for high-molecular-weight DNA extraction using a previously established protocol (Miller et al. 1988; Dias et al. 2021; Yang et al. 2021).

### Genome Assembly and Evaluation

We generated about 31 Gb long reads from Nanopore sequencing (PromethION P24) and 125 Gb paired-end short reads from Illumina (NovaSeq 6000). Long-read data were quality checked using NanoPlot v1.40 (Coster et al. 2018). Short-read data were quality trimmed with FastP v0.23.2 (Chen et al. 2018), and subsequently used to error correct the long reads with fmlrc2 (Mak et al. 2023). The error-corrected long read was used to assemble the genome, and the assembly was polished with short reads.

Prior to the genome assembly, we conducted a *k*-mer-based assessment, where the short reads were counted for the 21-mer using two independent *k*-mer counters, Jellyfish v2.3.0 (Marcais and Kingsford 2011) and Meryl within Merqury v1.3 (Rhie et al. 2020). The resulting 21-mer spectra were evaluated with the help of GenomeScope v2.0 (Ranallo-Benavidez et al. 2020; supplementary fig. S1, Supplementary Material online). We assembled the beetle genome from the error-corrected long reads using the Flye v2.9 (Kolmogorov et al. 2019) with default options. The Flye contigs were further joined by the program Longstitch v1.0.2 (Coombe et al. 2021) in “ntLink-arks” mode. The resulting contigs were called for consensus with medaka v1.6 (https://github.com/nanoporetech/medaka) and subsequently, polca corrected using short-read sequences (Zimin and Salzberg 2020). The polished assembly was screened for contaminants using Kraken v2.1.2 (Wood et al. 2019) against a redundant database of viruses, archaea, bacteria, and fungi. Based on the Kraken result, we removed 11 contigs identified as bacterial contaminants. The resulting assembly was labeled as draft assembly and analyzed further with the package getOrganelle v1.7.6.1 (Jin et al. 2020) to identify the mitochondrial contigs.

The draft assembly was checked for assembly statistics and completeness. We evaluated the assembly completeness against two orthologous databases, “Insecta” and “Endopterygota,” in the program BUSCO v5.4.4 (Manni et al. 2021). Then, we prepared a 21-mer library from high-quality short-read sequences using Merqury v1.3 (Rhie et al. 2020) and checked its distribution in the primary assembly to evaluate for the missing *k*-mers and duplications, if any. Based on the results from the evaluation steps, which indicated the occurrences of duplications (supplementary table S1 and fig. S2, Supplementary Material online), we corrected our primary assembly for haplotype preparation.

We separated the mitochondrial contig from our draft assembly to facilitate the downstream analyses. Therefore, we present the beetle genome assembly separately for the nuclear and mitochondrial parts. First, we identified the over-represented sequences in the nuclear assembly by mapping the error-corrected long reads to it and deriving a base-level read depth. Subsequently, with the assignment of appropriate cutoffs (-l 1, -m 21, -u 500) to the base-level coverage, we removed duplicates from the ends of the contigs by using the program Purgedups v2.22 (Guan et al. 2020). The purged nuclear assembly was screened for contaminants with NCBI FCS tool (https://github.com/ncbi/fcs) and subsequently, evaluated for assembly statistics and completeness as described earlier. Furthermore, we checked the assembly continuity in the repeat space by measuring the LAI—the intact proportion of genome-wide LTR (Ou et al. 2018).

To identify the phylogenetic position of *Z. bicolorata*, we performed a tree analysis by adding ten more Coleoptera genomes to the ingroup data and assigned two Lepidoptera *Bombyx mori* and *Lycia hirtaria* as outgroups. We reconstructed the tree from 669 complete and single-copy BUSCO proteins retrieved from all the samples in the data set. Protein sequences were aligned in MUSCLE v5.1 (Edgar 2022), and the low-confidence aligned sites were trimmed with trimAl v1.4 (Capella-Gutierrez et al. 2009). The concatenated alignment was then analyzed in IQ-TREE v2.2.3 (Minh et al. 2020) to predict the appropriate substitution model (Kalyaanamoorthy et al. 2017) and search for the maximum likelihood tree along with 1,000 ultrafast bootstrap (Hoang et al. 2017). The resulting tree was visualized and annotated in FigTree v1.4.4 (github.com/rambaut/figtree).

### Genome Annotation

To annotate the repeats in the assembled nuclear genome, we first prepared a repeat library by combining the repeats from the curated databases with de novo predicted ones. De novo repeat identification was carried out using the recently developed package Extensive de novo TE annotator (EDTA) v2.0.1, which uses diverse repeat identifying programs (Ou et al. 2019 and the references therein) in a curated manner to prepare a high-quality repeat library. As EDTA is currently limited to identifying certain repeat classes only (Ou et al. 2019), we, therefore, retrieved insect-specific additional repeat classes of SINE, LINE, satellite, and rDNA from the curated databases, SINEbase (Vassetzky and Kramerov 2013) and Dfam (Storer et al. 2021). Repeats from these databases were combined with those predicted from the EDTA and filtered for redundancies. The nonredundant library was then checked for any overlap with protein-coding sequences using the curated CDS data from another leaf-feeding beetle, *L. decemlineata* (OGS v1.2; Schoville et al. 2018). The final repeat library was used to annotate and soft mask the repeats in the assembly using RepeatMasker v4.1.5 (www.repeatmasker.org). The soft-masked assembly was further annotated for coding regions using BRAKER v3.0.3 (github.com/Gaius-Augustus/BRAKER). Prior to gene annotation, we used the curated protein sequences from *L. decemlineata* (OGS v1.2) and the arthropod-specific proteins from Orthodb v10 (Kriventseva et al. 2019) to train GeneMark and Augustus sequentially as implemented in BRAKER (Bruna et al. 2020). Following the training, Augustus performed the ab initio gene prediction (Lomsadze et al. 2005; Iwata and Gotoh 2012; Gotoh et al. 2014; Buchfink et al. 2015). Predicted genes were functionally annotated using eggnog-mapper v2.1.12 (Cantalapiedra et al. 2021) based on eggNOG orthology data (Huerta-Cepas et al. 2019).

### Mito Genome Assembly and NUMTs

Our draft genome assembly, prior to its haplotype correction, was searched for the occurrence of animal mitochondria using the package getOrganelle v1.7.6.1 (Jin et al. 2020), which recovered only one continuous fragment as the mitochondrial genome. The identified mitochondrial contig was then used to retrieve the long reads mapped to this region from a genome-wide mapping file. These recovered long-reads were assembled with Flye v2.8.1 (Kolmogorov et al. 2019) under the “meta” option and subsequently checked for contaminants by NCBI FCS (https://github.com/ncbi/fcs). We annotated the mitogenome using Mitos2 (Donath et al. 2019) and further characterized the identified CR for tandem repeats and palindromes using the package TRF v4.10 (Benson 1999) and IUPACpal (Alamro et al. 2021), respectively.

By performing a nucleotide “blast” of the mitochondrial genome against the nuclear genome, we identified the probable hits for the nuclear insertion of mitochondrial fragments (NUMTs). The identified hits were filtered for false positives by removing the hits if they matched only to the CR (low complex sequences) or were found nested within another hit or when the *E*-value >10^−6^ (following Richly and Leister 2004; Pamilo et al. 2007). The remaining hits were validated as NUMTs after we verified the read-level evidence confirming nuDNA-NUMT breakpoints. For this validation, we first retrieved long reads aligned to the entire length of each NUMT and spanning 100 bp flanking regions and computed NUMT-wise read depth (supplementary fig. S3, Supplementary Material online). One of the NUMTs was placed toward the start of a nuclear contig; hence, there was no upstream flanking region. We, therefore, limited the read depth computation in the given NUMT to the downstream flanking region only.

## Supplementary Material

evad188_Supplementary_Data

## Data Availability

Genome sequences generated during the study are archived in NCBI SRA, SRR25860156, and SRR25860157. This Whole Genome Shotgun project has been deposited at DDBJ/ENA/GenBank under the accession JAVJAQ00. The version described in this paper is version JAVJAQ01. Repeat and protein annotation files are submitted to Figshare and are available at dx.doi.org/10.6084/m9.figshare.24126162.
