# Supplementary material for: Nuclear and Mitochondrial Genome Assemblies of the Beetle, Zygogramma bicolorata, a Globally Important Biocontrol Agent of Invasive Weed Parthenium hysterophorus
Source: Genome Biol Evol. 2023 Oct 13;15(10):evad188. doi: 10.1093/gbe/evad188 (PMC10603765; doi:10.1093/gbe/evad188)
Supplement: evad188_Supplementary_Data [file evad188_supplementary_data.pdf]

Supplementary material for the title

Nuclear and mitochondrial genome assemblies of the beetle, *Zygogramma bicolorata*, a globally important biocontrol agent of invasive weed *Parthenium hysterophorus*

****

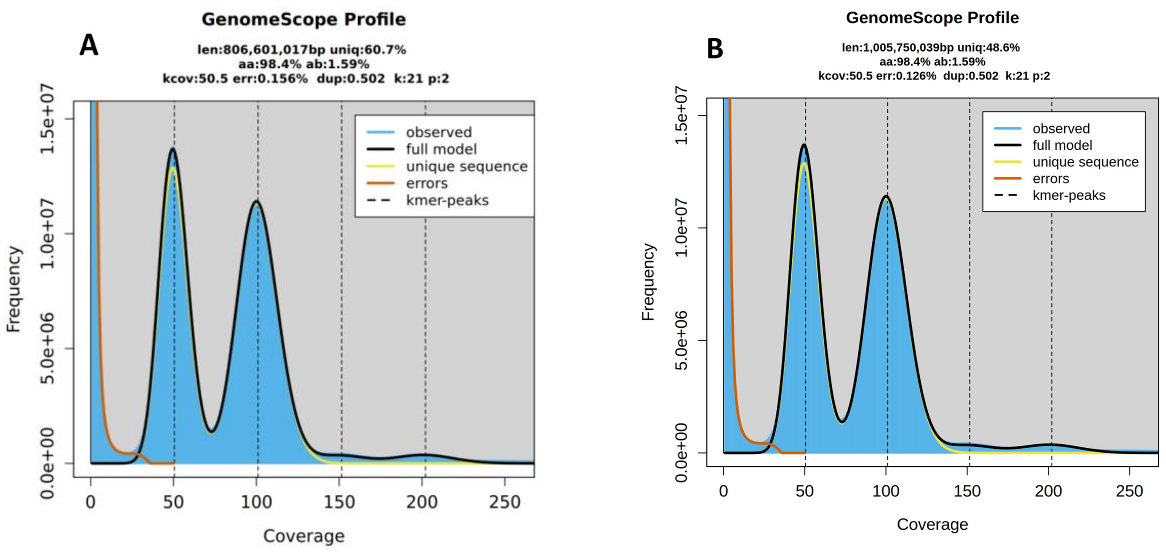

Figure S1: Results of GenomeScope profiling of 21-mer analysis of the short-read sequence in Jellyfish (A) and Meryl (B).

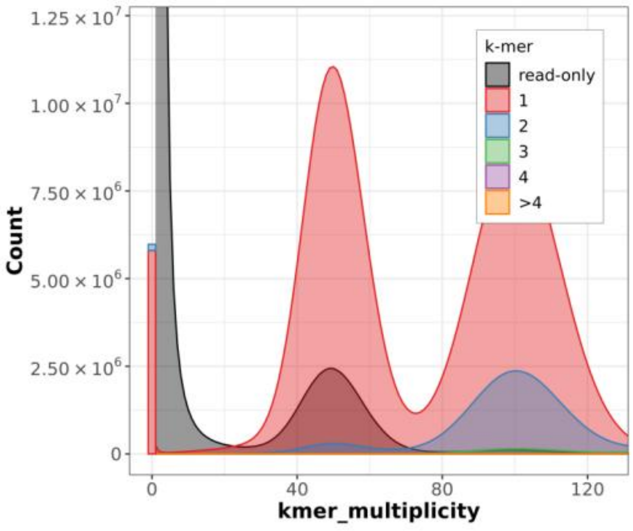

Figure S2: 21-mer distribution plot of illumina short-reads and the draft assembly (v1.1) from Merqury analysis. The plot indicates the occurrence of duplications in the draft assembly.

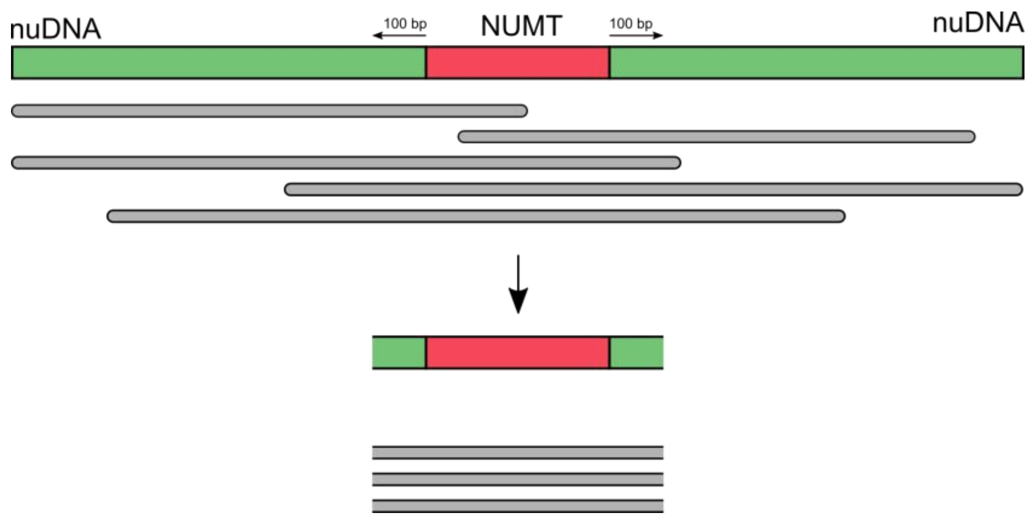

Figure S3: Diagram demonstrating the NUMT validation process. For each nuclear contig with identified NUMT, we filtered the mapped long-reads, from a genome-wide mapping file, to retrieve only those reads that span the NUMT along with 100 bp flanking regions.

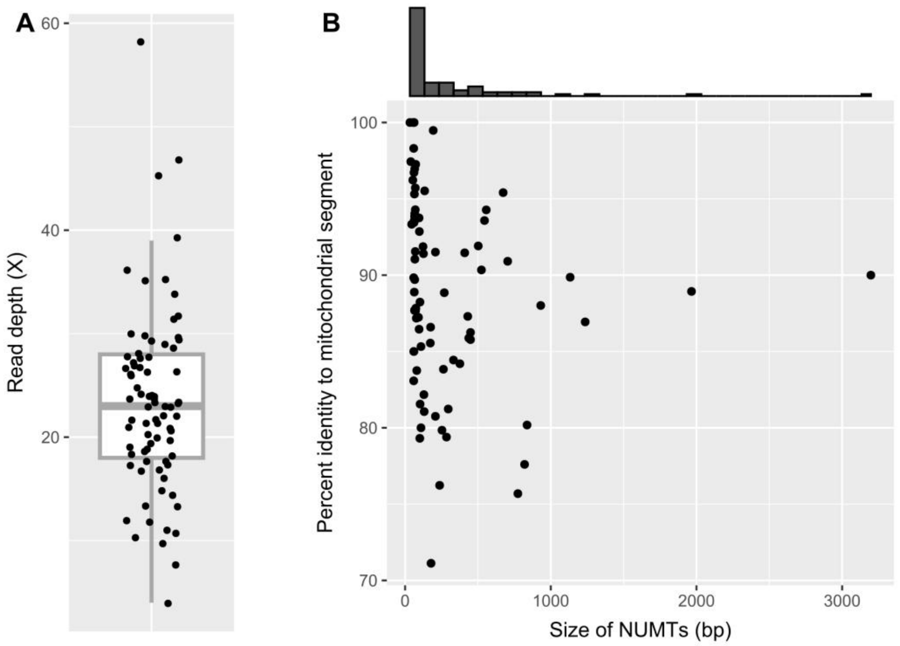

Figure S4: (A) Plot showing distribution of read depth of the validated NUMTs retrieved from the aligned long-read sequences. Read depth was computed following the steps depicted in Figure S3 above. (B) Distribution of blast similarity score of identified NUMTs against the mitochondrial genome. The bar graph on top depicts the frequency distribution of the blast score in relation to the NUMT size.

Table S1: BUSCO output of the draft assembly (v1.1), final nuclear assembly (v1.2), and only protein coding genes of the final nuclear assembly (v1.2).

	Assembly state →	draft v1.1	final v1.2	Protein-coding genes only
**Endopterygota _odb10 (2124)**	Complete	98.6 %	96.5%	89.8%
	► Single-copy	69.9 %	90.7%	77.2%
	► Duplicated	28.7 %	5.8%	12.6%
	Fragmented	0.6 %	1.0%	3.7%
	Missing	0.8 %	2.5%	6.5%
**Insecta_odb10 (1367)**	Complete	99.3 %	97.5%	90.9%
	► Single-copy	69.7 %	91.1%	77.5%
	► Duplicated	29.6 %	6.4%	13.4%
	Fragmented	0.1 %	0.6%	2.7%
	Missing	0.6 %	1.9%	6.4%

Table S2: Details of the genome annotation for repeat regions, coding regions and the intersection between them (for the final nuclear assembly v1.2).

Assembly size	935 913 224 bp
**Coding region**	
Number of genes	29 437
Length occupied: gene	210 376 192 bp
Length occupied: exon/CDS	36 578 457 bp
Length occupied: introns	197 943 307 bp
**Repeat region**	
Interspersed repeats	578 935 894 bp
► Retroelements	219 305 289 bp
► DNA transposons	283 217 119 bp
► Unclassified	76 413 486 bp
Satellites	7 790 bp
Simple repeats	6 337 738 bp
Low complexity	489 576 bp
**Intersection**	
Exons *vs.* repeats	3 067 500 bp
Introns *vs.* repeats	148 902 754 bp

Table S3: Comparison of assembly and annotation statistics between *Z. bicolorata* (for final nuclear assembly v1.2) and two other chrysomelid species.

	Zygogramma bicolorata **(this study)**	***Ophraella communa*** **(Bouchemousse et al. 2020)**	***Gonioctena quinquepunctata*** **(Lukicheva et al. 2021)**
GenBank accession	JAVJAQ000000000	GCA_902651945.1	GCA_018342105.1
Assembly level	Contig	Contig	Contig
Assembly size (Mb)	936	774	1732
Number of contig	6106	7003	10033
Longest contig (Mb)	16	--	3.03
Assembly N50 (Kb)	676	195	432
Assembly L50	293	925	1130
Gap (Ns) (%)	1.49	0	0
GC content (%)	35.1	31.9	34.6
BUSCO completeness (%) [Insecta_odb version]	97.5 [v10]	96 [v9]	95.5 [v9]
Repeat content (%)	62.7	58.2	72.9
Gene count	29437	75642	38493

Table S4: Assembly and annotation statistics of chrysomelid species from GenBank (accessed on 26 Aug 2023). Data was used to plot the Figures 2b, and 2c.

**species.name**	**genbank. accession**	**assembly. size**	**gene. count**	**percent. repeat**	**reference**
Ophraella communa	GCA_902651945.1	774 Mb	75642	58.2	Bouchemousse et al. 2020
Leptinotarsa decemlineata	GCA_000500325.2	642 Mb	16533	16.93	Schoville et al. 2018
Callosobruchus maculatus	GCA_900659725.1	1.01 Gb	21264	63.7	Sayadi et al. 2019
Gonioctena quinquepunctata	GCA_018342105.1	1732 Mb	38493	72.86	Lukicheva et al. 2021
Acanthoscelides obtectus	GCA_933228535.1	1103 Mb	38104	62.7	Immonen et al. 2023
Callosobruchus analis	GCA_947858975.1	1.9 Gb	38279	NA	Unpublished
Callosobruchus chinensis	GCA_944317955.1	1.4 Gb	41626	NA	Unpublished
Diabrotica balteata	GCA_918026665.1	1.6 Gb	13810	NA	Unpublished
Diabrotica virgifera virgifera	GCA_917563875.2	2.5 Gb	24968	NA	Unpublished
Phaedon cochleariae	GCA_918026855.4	870 Mb	13141	NA	Unpublished
Phyllotreta striolata	GCA_918026865.1	132 Mb	11862	NA	Unpublished
Psylliodes chrysocephala	GCA_927349885.1	1.2 Gb	15768	NA	Unpublished
Diorhabda carinulata	GCA_026250575.1	415 Mb	13668	NA	Unpublished
Diorhabda sublineata	GCA_026230105.1	456 Mb	13047	NA	Unpublished
Zygogramma bicolorata	JAVJAQ000000000	936 Mb	29437	62.7	This study

In cases of discrepancy, data from the original publication was preferred, when available, except in *L. decemlineata*, where data was majorly compiled from NCBI-GenBank. Information for *Z. bicolorata* is for nuclear assembly only.

1. Bouchemousse S, Falquet L, Müller-Schärer H. 2020. Genome assembly of the ragweed leaf beetle: a step forward to better predict rapid evolution of a weed biocontrol agent to environmental novelties. Genome Biol. Evol. 12: 1167-1173.
2. Immonen E, et al. 2023. Experimental life history evolution results in sex-specific evolution of gene expression in seed beetles. *Genome Biol. Evol.* 15: evac177.
3. Lukicheva S, Flot JF, Mardulyn P. 2021. Genome assembly of the cold-tolerant leaf beetle *Gonioctena quinquepunctata*, an important resource for studying its evolution and reproductive barriers between species. *Genome Biol. Evol.* 13:evab134.
4. Sayadi A, et al. 2019. The genomic footprint of sexual conflict. *Nat. Ecol. Evol.* 3:1725-1730.
5. Schoville SD, et al. 2018. A model species for agricultural pest genomics: the genome of the Colorado potato beetle, *Leptinotarsa decemlineata* (Coleoptera:Chrysomelidae). *Sci. Rep.* 8:1931.

Table S5: Data on genome size of chrysomelid species from GenBank (accessed on 26 Aug 2023). For multiple assemblies per genome, the largest one was considered. Data was used to plot the Figure 2a.

**species.genome**	**genbank.accession**	**size.bp**
Acanthoscelides obtectus	GCA_933228535.1	1103332300
Agelastica alni	GCA_950111635.1	692269472
Bruchidius siliquastri	GCA_949316355.1	375568116
Callosobruchus analis	GCA_947858975.1	1918300402
Callosobruchus chinensis	GCA_944317955.1	1402444044
Callosobruchus maculatus	GCA_900659725.1	1007816681
Chrysolina americana	GCA_958502065.1	980572297
Chrysolina haemoptera	GCA_958298965.1	718304979
Chrysolina oricalcia	GCA_944452925.2	1423436458
Chrysomela aeneicollis	GCA_029955535.1	646738179
Crepidodera aurea	GCA_949320105.2	508956902
Crioceris asparagi	GCA_958507055.1	639283977
Cryptocephalus moraei	GCA_946251905.1	500544478
Diabrotica balteata	GCA_918026665.1	1607257046
Diabrotica virgifera virgifera	GCA_917563875.2	2533404242
Diorhabda carinata	GCA_029229535.1	449895081
Diorhabda carinulata	GCA_026250575.1	415369604
Diorhabda elongata	GCA_026230145.1	485337276
Diorhabda sublineata	GCA_026230105.1	456219552
Galerucella calmariensis	GCA_910591675.1	588272243
Galerucella pusilla	GCA_910591695.1	513237794
Galerucella tenella	GCA_910591685.1	460592984
Gonioctena quinquepunctata	GCA_018342105.1	1731947787
Hermaeophaga mercurialis	GCA_951812935.1	479394699
Leptinotarsa decemlineata	GCA_024712935.1	1008323064
Leptinotarsa defecta	GCA_015342085.1	623388812
Leptinotarsa haldemani	GCA_015342125.1	549178770
Leptinotarsa juncta	GCA_015342185.1	578212017
Leptinotarsa lineolata	GCA_015342045.1	647430020
Leptinotarsa peninsularis	GCA_015342205.1	558697164
Leptinotarsa rubiginosa	GCA_015342145.1	566666884
Leptinotarsa texana	GCA_015342105.1	640408317
Leptinotarsa tumamoca	GCA_015342025.1	578677183
Leptinotarsa undecemlineata	GCA_015342065.1	639105767
Lochmaea capreae	GCA_949126875.1	534673670
Lochmaea crataegi	GCA_947563755.1	891283075
Neocrepidodera transversa	GCA_963243735.1	671299900
Ophraella communa	GCA_902651945.1	774411302
Phaedon cochleariae	GCA_918026855.4	869734947
Phyllotreta cruciferae	GCA_917563865.1	135120912
Phyllotreta striolata	GCA_918026865.1	132261818
Plagiodera versicolora	GCA_025400015.1	157911003
Psylliodes chrysocephala	GCA_927349885.1	1181805965
Zygogramma bicolorata	JAVJAQ000000000	935913224

Genome size of *A. obtectus* was updated from the original publication, Immonen et al. 2023. Information for *Z. bicolorata* is for nuclear assembly only.
